# Blood Stasis Syndrome Accelerates the Growth and Metastasis of Breast Cancer by Promoting Hypoxia and Immunosuppressive Microenvironment in Mice

**DOI:** 10.1155/2022/7222638

**Published:** 2022-06-07

**Authors:** Lu Jin, Biqiang Tang, Xia Liu, Weiye Mao, Linying Xia, Yueguang Du, Bing Ji, Qiyang Shou, Huiying Fu

**Affiliations:** ^1^Second Clinical Medical School, Zhejiang Provincial Key Laboratory of Sexual Function of Integrated Traditional Chinese and Western Medicine, Zhejiang Chinese Medical University, Hangzhou 310053, China; ^2^Huzhou Hospital of Traditional Chinese Medicine, Affiliated Zhejiang Chinese Medicine University, Huzhou, 313003, China; ^3^Nanxun People's Hospital, Huzhou 313003, China; ^4^School of Pharmacy, Zhejiang Chinese Medical University, Hangzhou 310005, China; ^5^School of Basic Medicine, Zhejiang Chinese Medical University, Hangzhou 310005, China

## Abstract

Blood stasis syndromes (BSSs) are closely related to the occurrence and development of tumors, although the mechanism is still unclear. This study was aimed at exploring the effect and mechanism underlying different BSSs on tumor growth and metastasis. We established four BSS mouse models bred with breast cancer: qi deficiency and blood stasis (QDBS), cold coagulation blood stasis (CCBS), heat toxin and blood stasis (HTBS), and qi stagnation and blood stasis (QSBS). The results showed that microcirculation in the lower limb, abdominal wall, and tumor in situ decreased by varying degrees in the BSS groups. In addition, BSS promoted tumor growth and lung metastasis. The ratio of regulatory T cells in the tumor microenvironment was downregulated. Moreover, hypoxia-inducible factor 1-*α*, Wnt1, *β*-catenin, vascular endothelial growth factor, and Cyclin D1 levels increased in the tumors of BSS mice. In conclusion, BSS not only promoted the formation of a hypoxic and immunosuppressive microenvironment but also promoted the neovascularization.

## 1. Introduction

According to traditional Chinese medicine (TCM), blood stasis syndrome (BSS) is usually described as unsmooth blood flow in vessels or the withdrawal of storage outside the blood overflow [1]. Depending on the different pathogenesis and etiology of blood stasis syndrome, BSSs can be divided into qi deficiency and blood stasis (QDBS), cold coagulation and blood stasis (CCBS), heat toxin and blood stasis (HTBS), qi stagnation and blood stasis (QSBS), and other subtypes [2]. In addition, TCM holds that stagnation of qi and blood stasis are the basic cause of cancer. Tang Rongchuan, a famous doctor in the Qing Dynasty, put forward in his Treatise on Blood Troubles that “blood stasis between meridians and viscera is cancer”. This also suggests that BSS is closely related to cancer.

Based on the similarity of pathogenesis, clinical manifestations, and diagnostic criteria between a hypercoagulable state and blood stasis syndrome, TCM ascribes the hypercoagulable state in modern medicine to the category of BSSs; “blood stasis” is its main pathogenic factor and pathological product [3]. Hypercoagulable state, also known as a prethrombotic state or a thrombosis tendency, refers to the disorder of hemostasis, coagulation, and anticoagulant system in the body caused by the pathological changes of blood vessels, blood, and blood flow components [4]. The hypercoagulable state of the body can promote the growth and metastasis of tumors, which can break the balance of the body's coagulation, anticoagulation, and fibrinolysis systems to again cause a hypercoagulable state, thus perpetuating a vicious cycle [5]. More than 100 years ago, Armand Trousseau, a professor in clinical medicine, observed that in some patients, cancer was accompanied by blood hypercoagulability [6]. When the blood is in a hypercoagulable state, the platelet count increases and vascular endothelial growth factor (VEGF), which secretes from platelets, can promote angiogenesis [7]. New vessels provide nutrition for tumor cells and promote the growth and metastasis of tumors. In addition, platelets gather around tumor cells, forming a physical barrier to protect tumor cells from natural killer cell-mediated lysis [8, 9], to limit their exposure to shear stress, and to facilitate their adhesion to the endothelium [10–12]. Therefore, a hypercoagulable state is closely related to the occurrence and development of tumors.

Studies have shown that the hypercoagulable state, or blood stasis, in patients with tumors is closely related to the formation of a tumor immunosuppressive microenvironment [1–3]. Once blood stasis occurs, it can inhibit the body's antitumor immune function in multiple steps and promote the occurrence and development of tumors [13]. For example, it has been shown that blood stasis in tumors is the main cause of ischemia and hypoxia in the tumor microenvironment [14]. Nevertheless, how different BSSs affect the tumors and tumor microenvironment is still unclear.

Breast cancer is currently the most common malignancy in women worldwide. Distant recurrence and metastasis are the main causes of death in breast cancer patients. According to traditional Chinese medicine, blood stasis syndrome is one of the important pathological mechanisms of the occurrence and development of breast cancer [15]. In this study, we used the 4T1-Luc breast cancer in situ model, which is a good tumor model focusing on the immune system [16]. 4T1 breast cancer cells spontaneously transfer to the lungs after entering the blood, it can be used to observe the effect of blood stasis on tumor growth and metastasis. This study was aimed at observing the effects of different BSSs on breast cancer by establishing QDBS, CCBS, HTBS, and QSBS mouse models with breast cancer and exploring the mechanism by which they promoted tumor activity.

## 2. Methods

### 2.1. Cell Culture

4TI-Luc cells were cultured in RPMI-1640 media (Thermo Scientific, USA) supplemented with penicillin (100 IU/mL), streptomycin (100 *μ*g/mL), and 10% fetal bovine serum (Thermo Scientific, USA). The cells were incubated in a humidified atmosphere of 5% CO_2_ at 37°C.

### 2.2. Animal Experiments

Forty BALB/c female mice (8 weeks old, weighing 20–22 g) were purchased from the Shanghai Laboratory Animal Center (Shanghai, China) and kept in a specific pathogen-free mouse breeding room with a controlled temperature of 24 ± 1°C. The mice were provided free access to food. The animal experiment was reviewed and approved by the Committee on the Ethics of Animal Experiments of Zhejiang Chinese Medical University (Number of resolution: ZSLL-2018–053).

After 7 days of adaptation, the BALB/c mice were randomly divided into five groups (*n* = 8 per group) as follows: (1) control group; (2) QDBS group: the mice were subjected to exhaustion by swimming [17]; (3) QSBS group: adrenaline (0.08 mg/kg) was injected once a day for 7 days [18]; (4) CCBS group: the mice were soaked in ice water at 0–1°C for approximately 10 min daily for 44 days [19]; and (5) HTBS group: carrageenan (10 mg/kg) was subcutaneously injected on the first day of modeling, and bacterial lipopolysaccharide (LPS) (50 *μ*g/kg) was injected intravenously 16 h later; they were injected every other day for 3 weeks [20]. For the QDBS group, exhaustive swimming modeling was conducted once a day with starvation diet modeling for 3 weeks. The standard for exhaustive swimming was as follows: swimming movement was maladjusted, the tip of the nose was submerged in water, and the body sank for more than 10 s without surfacing. For the HTBS group, endotoxin, which is a hot and poison evil, can make blood sticky and burn blood vessels, then caused adverse blood flow, as a result, the blood stasis occurred [21]. During the experiment, all groups freely ate a high-fat and high-sugar diet (a high-fat and high-sugar diet increases the blood viscosity and accelerates the formation of blood stasis) [22], except the control group.

One week after the blood stasis models were established according to the above methods, 4T1-Luc cells (5 × 10^5^) in 100 *μ*L phosphate-buffered saline were injected into the bottom-right second mammary fat pad. The mice were weighed twice a week, and tumor volumes were measured by a vernier caliper. Tumor volume was calculated as *V* = (length × width^2^)/2. Bioluminescence imaging was performed at weeks 2 and 4 after tumor cell injection. On day 34 of making BSS models, we used laser speckle to detect the microcirculation blood flow in the abdominal wall, limbs, and tumor sites. Then, the mice were sacrificed with CO_2_ asphyxiation for tumor and lung collection.

### 2.3. Bioluminescence Imaging

Before imaging, the mice were intraperitoneally injected with luciferase substrate (150 mg/kg, Gold Biotechnology, St. Louis, MO, USA). The mice were anesthetized with a mixture of 4% isoflurane and 30% oxygen for 15 min and then placed on the Xenogen IVIS 200 imaging system (Caliper Life Sciences, Hopkinton, MA, USA) to collect in vivo images. LT Living Image 4.3 software was used for data analysis.

### 2.4. Laser Speckle Imaging

All mice were fixed in the supine position under isoflurane respiratory anesthesia. Blood flow was detected with the moor FLPI-2 speckle blood flow imaging system (Moor Instruments Ltd., UK) in multi-image measurement mode. Images were saved and analyzed using the moor FLPI-2 review software (version 2.0).

### 2.5. Flow Cytometry Assay

The tumor tissues were put into a 6-well plate containing 1.5 mL of harvest media (RPMI 1640, 2% heat-inactivated fetal calf serum, 1% penicillin/streptomycin), ground with a grinding rod, then filtered through 70 *μ*m filter membrane to acquire single-cell suspension. Then, cells were incubated with surface antibodies at 25°C for 30 min in the dark. The anti-mouse antibodies were as follows: anti-CD45 PE-Cy7 (1 : 500, cat: No.103114, BioLegend), anti-CD3 BV605 (1 : 500, cat: No.563004, BD Pharmingen), anti-CD4 FITC (1 : 500, cat: No.553046, BD Pharmingen), anti-CD8 APC-H7 (1 : 400, cat: No.560182, BD Pharmingen), anti-F4/80 PE (1 : 500, cat: No.565410, BD Pharmingen), anti-CD25 BV605 (1 : 500, cat: No. 563061, BD Pharmingen), anti-CD11b AF700 (1 : 500, cat: No. 557960, BD Pharmingen), anti-CD86 BV510 (1 : 500, cat: No. 563077, BD Pharmingen), and anti-CD206 AF647 (1 : 200, cat: No. 565250, BD Pharmingen). To detect regulatory T cell (Treg) population in tumor microenvironment, cells were fixed with Cytofix/Cytoperm (BD Biosciences) for 60 min at 4°C before intranuclear staining, then washed with perm/wash buffer twice, and centrifuged at 350 × g at 2-8°C for 6 min. Cells were incubated with anti-Foxp3 PE (1 : 100, cat: No.560408, BD Pharmingen) antibody at 25°C for 50 min in the dark, washed, and centrifuged again. Cells were suspended with stain buffer; then, samples were captured using FACS Canto II Cytometry (BD Biosciences, USA), and the data were analyzed with FlowJo.

### 2.6. Hematoxylin-Eosin Staining of Lung Tissues

Lung tissues were fixed using 10% formaldehyde for 24 h, then dehydrated and embedded in paraffin, sliced into 4 *μ*m sections, and stained with hematoxylin and eosin according to the protocol. Sections were imaged using a NanoZoomer Digital Slide Scanner (NDP; Nikon, Tokyo, Japan) and analyzed in NDP.view.

### 2.7. Western Blot Analysis

100 mg tumor tissue was lysed in 400 *μ*L radioimmunoprecipitation assay lysis buffer (Shanghai Biyuntian Biotechnology Co., Ltd., Hangzhou, China) containing 1% phenylmethanesulfonyl fluoride. The lysates were centrifuged at 12,000 rpm for 10 min at 4°C, and the supernatant was collected. Protein concentration was detected using a BCA protein concentration determination kit. Subsequently, samples were separated on 10% sodium dodecyl sulphate/polyacrylamide gel electrophoresis gel, transferred to a polyvinylidene fluoride (PDVF) membrane, and blocked with 5% skim milk. Primary antibodies were diluted according to the manufacturer's recommendations and incubated with the membrane overnight at 4°C. The primary antibodies used were as follows: *β*-actin (1 : 1000, HuaAn Biotechnology Co., Ltd. China), VEGF (1 : 1000, Abcam, UK), *β*-catenin, hypoxia-inducible factor 1-alpha (HIF1-*α*), Wnt1, and Cyclin D1 (1 : 1000, Cell Signaling Technology, USA). After the membranes were washed three times with triethanolamine-buffered saline for 10 min each, blots were incubated with the corresponding secondary antibody (1 : 10000, Cell Signaling Technology, USA) at room temperature for 2 h. The protein bands were visualized with Millipore Western Blot HRP chemiluminescence solution (MilliporeSigma Corporation, Billerica, MA, USA) and scanned by ultrasensitive chemiluminescence imaging system (Bio-Techne, USA). AlphaView-FluorChem FC3 3.4.0.0 software (ProteinSimple, San Jose, California, USA) was used to quantify the band intensity and normalized to *β*-actin.

### 2.8. Statistical Analysis

Statistical analysis was performed using the SPSS 25.0 software. All data were expressed as means ± standard errors of the mean. Data were analyzed by one-way analysis of variance, with *P* < 0.05 indicating a statistically significant difference.

## 3. Results

### 3.1. Evaluation of the Blood Stasis Syndrome Model

**Figure 1:**
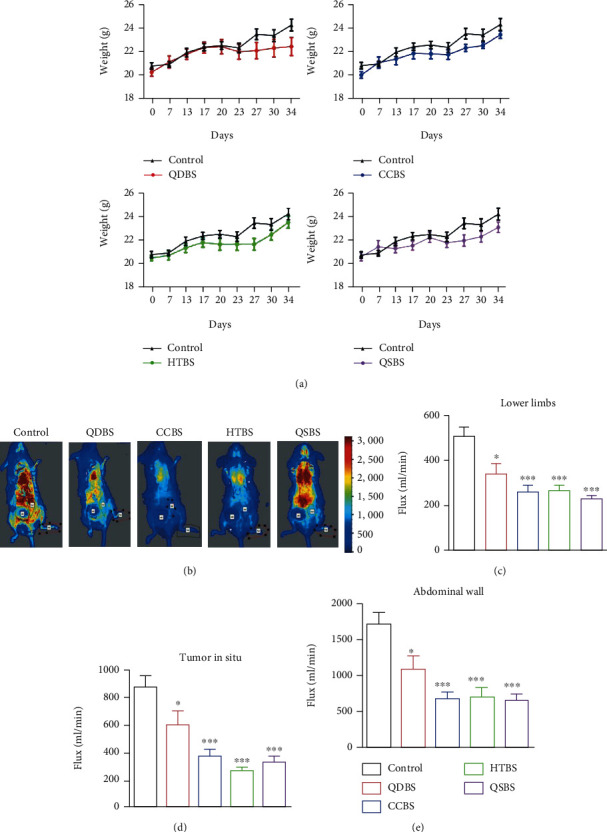
Body weight and microcirculation of mice. (a) Weight of the mice in each group. Tumor cells were injected on day 7 and started growing on day 13. We weighed the mice on days 0, 7, 13, 20, 23, 23, 27, 30, and 34 after moulding. (b) Representative image of microcirculation in mice from each group. (c) Quantitative statistics of microcirculation in the lower limbs. (d) Quantitative statistics of tumor microcirculation. (e) Quantitative statistics of abdominal wall microcirculation. ^∗^*P* < 0.05, ^∗∗^*P* < 0.01, and ^∗∗∗^*P* < 0.001, vs. control.

After the BSSs model was established, the growth rate by weight in the BSS groups was slower than that in the control group (Figure 1(a)). In order to observe the microcirculation blood flow of mice, on day 34 of making models, we used laser speckle to detect the microcirculation blood flow in the abdominal wall, limbs, and tumor sites. The results showed that the microcirculation of the lower limbs was significantly lower in the BSS groups (Figures 1(b) and 1(c)). Moreover, microcirculation of the abdominal wall and tumor in situ in the HTBS and QSBS groups was also significantly lower than that of the control group (Figures 1(d) and 1(e)). Decrease of body weight and microcirculation blood flow indicated that the blood stasis model was successful.

### 3.2. Blood Stasis Syndrome Promoted Tumor Growth in Mice

**Figure 2:**
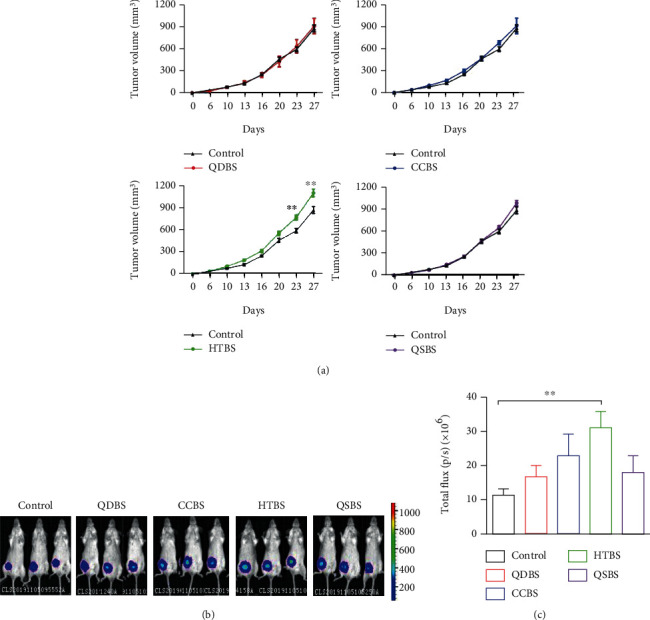
Influence of blood stasis syndrome on breast cancer in situ. (a) Tumor volumes were compared between the blood stasis syndrome and control groups. We took the day which tumor cells were injected as day 0, and the tumor grew on day 6. (b) Representative luciferase imaging of mice in the second week after tumor inoculation. (c) Quantitative statistics of tumor in situ fluorescence image. ^∗^*P* < 0.05 and ^∗∗^*P* < 0.01, vs. control.

One week after the blood stasis models were established, 4T1-Luc cells were injected. Tumors began to grow at 6 days of injection; then, we used a vernier caliper to measure the tumor diameter and calculated the tumor volume every week. Compared with the control group, the volume of in situ tumors in the QDBS, CCBS, and QSBS groups grew faster, but there was no significant difference. The tumor volume in the HTBS group had a significant difference when compared with the control group (Figure 2(a)). The results from the bioluminescence imaging also indicated that blood stasis syndrome promoted the tumors in the mice; specifically, the HTBS was the most affected (Figures 2(b) and 2(c)).

### 3.3. Blood Stasis Syndrome Promoted Lung Metastasis in Mice

**Figure 3:**
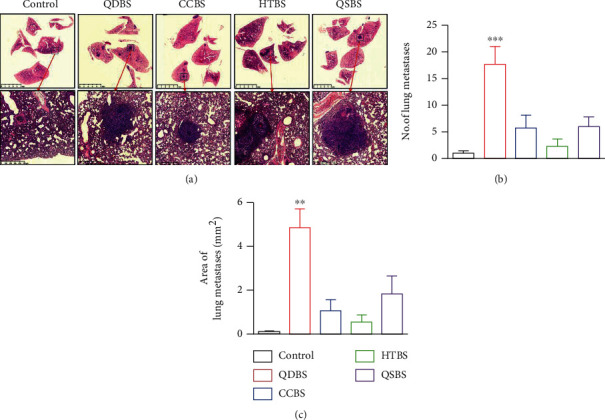
Effect of blood stasis syndrome on lung metastasis in mice. (a) Representative image of pathological hematoxylin and eosin staining in the lung tissue of the control and blood stasis syndrome groups. Scale bar: 5 mm and 500 *μ*m. (b) Quantitative statistics of the metastatic lesion number in the lung tissue. (c) Quantitative statistics of the metastatic lesion area in the lung tissue. ^∗∗^*P* < 0.01 and ^∗∗∗^*P* < 0.001, vs. control.

In the fifth week of the experiment, we stained the lung tissue with hematoxylin and eosin staining and observed a different enhancement of lung metastasis in the BSS group compared with that in the control group (Figure 3(a)). The area and number of nodules in lung were significantly increased in QDBS group (Figures 3(b) and 3(c)).

### 3.4. Blood Stasis Syndrome Promoted the Formation of a Tumor Immunosuppressed Microenvironment

**Figure 4:**
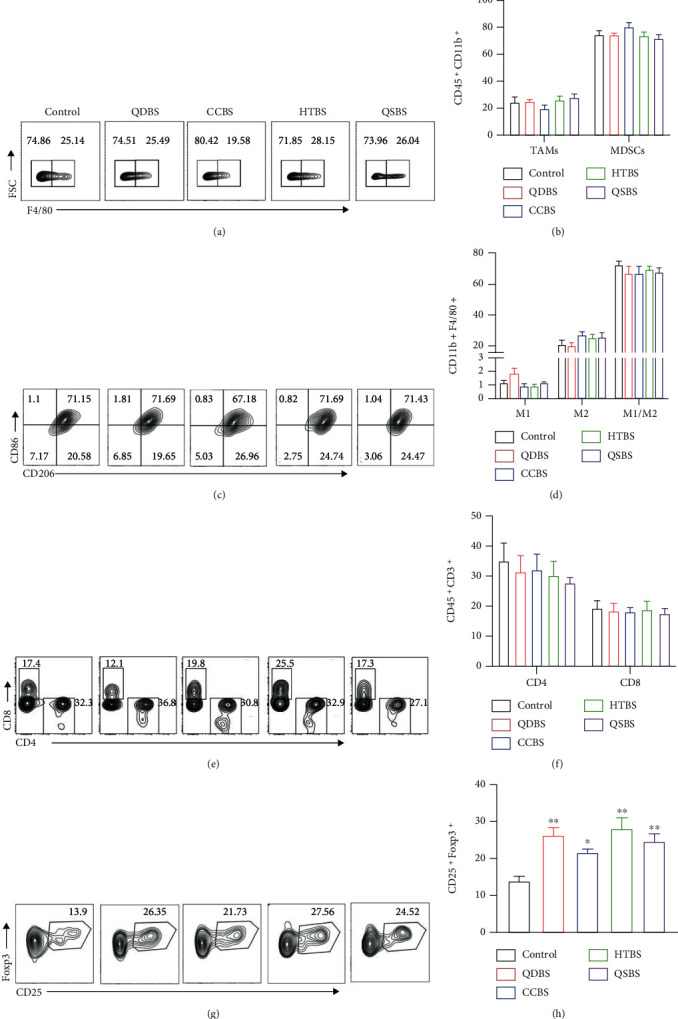
Effect of blood stasis syndrome on the tumor microenvironment. (a, b) Flow cytometry showing the percentage of myeloid-derived suppressor cells (CD11b^+^F4/80^−^) and tumor-associated macrophages (TAMs) (CD11b^+^F4/80^+^) in the tumor tissues. (c, d) Flow cytometry showing the percentage of M1-TAMs (CD86^+^CD206^−^) and M2-TAMs (CD86^−^CD206^+^) in the tumor tissues. (e, f) Flow cytometry showing the percentage of CD8^+^ T cells in the tumor tissues. (g, h) Flow cytometry showing the percentage of regulatory T cells (CD4^+^CD25^+^FoxP3^+^) in the tumor tissues. ^∗^*P* < 0.05, ^∗∗^*P* < 0.01, and ^∗∗∗^*P* < 0.001, vs. control.

Next, we further studied whether different blood stasis syndrome types were accompanied by changes in the tumor microenvironment. Flow cytometry was used to detect the ratio of myeloid-derived suppressor cells (MDSCs), tumor-associated macrophages (TAMs), T cells, and regulatory T cell (Treg) in tumor tissues. As shown in Figure 4, the expression of MDSCs (CD45^+^CD11b^+^F4/80^−^), TAMs (CD45^+^CD11b^+^F4/80^+^), M1-TAMs (CD45^+^CD11b^+^F4/80^+^CD86^+^CD206^−^), and M2-TAMs (CD45^+^CD11b^+^F4/80^+^CD86^−^CD206^+^) in the BSS groups was not significantly different compared with that in the control group (Figures 4(a)–4(d)). The population of CD4^+^ and CD8^+^ T cells was reduced in the BSS groups (Figures 4(e) and 4(f)), but there were no statistically significant differences. Moreover, the Treg population was significantly increased in the blood stasis group (Figures 4(g) and 4(h)).

### 3.5. Blood Stasis Syndrome Upregulates the Expression of VEGF and Cyclin D1 through Hif1-*α*/Wnt/*β*-Catenin Signaling Pathway

**Figure 5:**
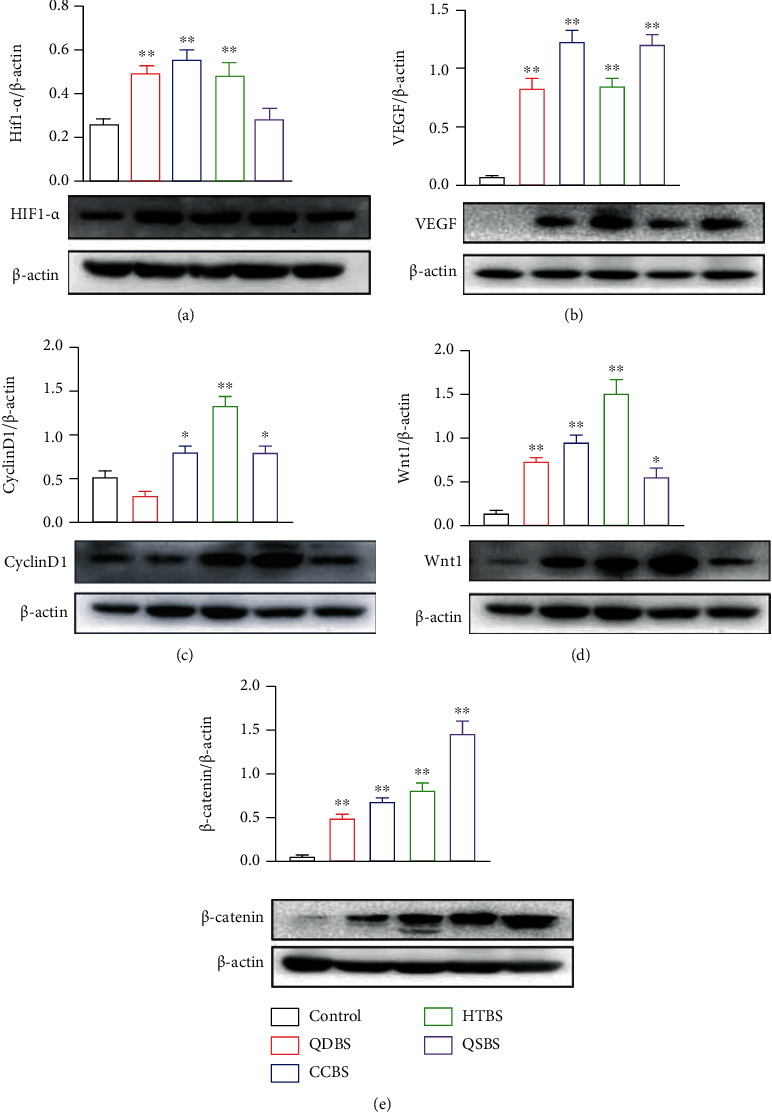
Expression of hypoxia-inducible factor 1-alpha (HIF-1*α*), vascular endothelial growth factor (VEGF), Cyclin D1, Wnt1, and *β*-catenin in tumors of the blood stasis syndrome groups. (a) Expression of HIF-1*α* in tumor tissues. (b) Expression of VEGF in tumor tissues. (c) Expression of Cyclin D1 in tumor tissues. (d) Expression of Wnt1 in tumor tissues. (e) Expression of *β*-catenin in tumor tissues. ^∗^*P* < 0.05, ^∗∗^*P* < 0.01, and ^∗∗∗^*P* < 0.001, vs. control.

In order to further analyze the mechanism of the tumor microenvironment affecting the growth and metastasis of breast cancer with blood stasis syndrome, we used western blotting to detect the changes of related protein indicators in tumor tissues. As shown in Figure 5, HIF1-*α*, VEGF, Cyclin D1, Wnt1, and *β*-catenin expression in the tumor was detected by Western blotting. The protein levels of HIF1-*α* in QDBS, CCBS, and HTBS were significantly higher than those in the control mice, although there was no significant difference in the QSBS group (Figure 5(a)). The expression of VEGF protein in tumors markedly increased in the BSS groups (Figure 5(b)). Cyclin D1 levels in the CCBS, HTBS, and QSBS groups increased significantly, while they decreased slightly in the QDBS group (Figure 5(c)). Levels of Wnt1 and *β*-catenin were significantly increased in all the experimental groups (Figures 5(d) and 5(e)). These results suggest that blood stasis syndrome may regulate the growth and metastasis of mouse breast cancer through the HIF1-*α*/Wnt/*β*-catenin signaling pathway.

## 4. Discussion

Blood stasis causes various pathological changes, such as inflammation, necrosis, edema, hyperplasia, exudation, sclerosis, and degeneration [23]. Many diseases can be further divided into different blood stasis syndrome subtypes based on TCM diagnosis. And previous studies explored the different blood stasis syndrome subtypes' effect on disease. For example, clinically, primary dysmenorrhea was divided into coagulation and blood stasis mode (CCBSP) and qi stagnation and blood stasis mode (QSBSP) to evaluate the therapeutic effect of electroacupuncture [24]. Urinary metabolomic profiling revealed significantly different metabolites with QSBS or QDBS subtypes of coronary heart disease, suggesting that these subtypes are different to one another at the metabolic level [25]. Bulbar conjunctival microcirculation in rabbits of five blood stasis syndrome subtypes (HTBS, QDBS, QSBS, CCBS, external injury, and blood stasis syndrome) was different, which could reflect different pathological mechanisms of rabbit balloon conjunctiva with BSSs [26]. Until now, the effect mechanism of blood stasis syndrome on tumor is still unclear. Therefore, we explored the mechanisms by which different blood stasis syndromes affected tumor growth and metastasis. In this study, we established four blood stasis models, which were confirmed by the changes in body weight and microcirculation. We found that BSSs promote tumor growth and metastasis; moreover, different BSSs have varying impacts on the tumor process. The HTBS group promoted the fastest tumor growth in the mice, and the QDBS group had the most obvious lung metastasis.

BSSs occur due to blood being in a hypercoagulable state, blood stasis resistance, and unsmooth veins [1]. Blood circulation is responsible for the exchange of substances with the cells supplied by the capillary network, which provides nutrients and oxygen for the survival of various cells of the body, and removes the products of cell metabolism [7]. When blood circulation is impaired, the body will become hypoxic [14]. Blood of patients with a malignant tumor is usually in a hypercoagulable state [4, 22]; they experience blood stasis and internal obstruction, and their veins are unsmooth, which leads to a local microcirculation disturbance and aggravates the ischemia and hypoxic state.

Immunosuppression is closely related to the growth and metastasis of breast cancer [27]. However, it is still unclear how cell subsets form in the immune-tolerant microenvironment of a tumor in a patient with BSSs. Different cytokines interact with each other to induce functional changes in tumor cells and immune cells and cause malignant biological behaviors such as tumor cell proliferation, invasion, and metastasis by forming a dynamic and complex tumor immune microenvironment [28]. It is well known that CD4^+^CD25^+^Foxp3^+^ Tregs suppress anticancer immunity [29]. Tregs mainly migrate to inflammatory sites and suppress various types of effector lymphocytes, including CD4^+^ T helper cells and CD8^+^ cytotoxic T lymphocytes [30]. Targeted inhibition of STAT3 expression on Treg cells improved the control of local and distant disease progression and enhances NK-mediated metastasis immune surveillance [31]. Moreover, high expression of Treg promotes lymph node metastasis and reduces immunotherapy efficacy in patients with tumors with liver metastases [32, 33]. Thus, when Tregs send signals that repress the immune response, they hamper effective antitumor and metastasis immune responses in tumor-bearing hosts. In our research, we found that the tumor suppressor microenvironment was more serious with BSSs, the ratio of CD4^+^ and CD8^+^ was slightly downregulated, and the ratio of Tregs was significantly upregulated, especially for the HTBS and QDBS groups, the proportion of Treg was highest. So, we found the HTBS group promoted the fastest tumor growth in the mice, and the QDBS group had the most obvious lung metastasis.

The study of Chen and Shi, who are long-termresearchers of BSSs, confirmed that the pathophysiological nature of bloodstasis is hypoxia. [34]. In the hypoxic microenvironment, tumor cells not only can continue to survive and grow but also regulate vessel growth, escape hypoxic stress injury, and adapt to the hypoxic environment, which is crucial for the growth and metastasis of tumors [35–37]. HIF1-*α* could activate the Wnt/*β*-catenin signal under hypoxia [38]. The Wnt/*β*-catenin signaling pathway can regulate proliferation, differentiation, apoptosis, and carcinogenesis of cells. VEGF and Cyclin D1 are downstream of the Wnt/*β*-catenin pathway [39]. Cyclin D1 regulates the cell cycle [40], and activation of Cyclin D1 induces abnormal proliferation of tumor cells [41]. VEGF is a vascular endothelial cell growth factor that can improve the permeability of blood vessels and provide material support for the growth and metastasis of tumor cells [42]. Blood vessels in the tumor tissue are highly abnormal. With the continuous production of VEGF, tumor blood vessels become curved, expanded, and unevenly distributed. The coverage of pericytes is low, and the connection between endothelial cells is loose, which enhances the permeability of blood vessels, increases the pressure of tissue stromal fluid, flattens tumor blood vessels under high stromal pressure, and reduces tumor perfusion, resulting in a hypoxic tumor microenvironment [43]. In our experiment, Western blot results showed that the expression of HIF-1*α*, Wnt, *β*-catenin, VEGF, and Cyclin D1 in the BSS group increased significantly. Therefore, BSSs promote the regeneration of tumor blood vessels. Tumor cells are more likely to transfer to other organs through blood vessel walls and vessels, thereby promoting lung metastasis.

The HTBS group was modelled with the continuous stimulation of LPS. Bacterial endotoxin LPS is a heat source and inflammatory stimulating factor that can induce chronic inflammation in the body. In our experiment, LPS was injected intravenously every other day for 3 weeks, the long-term stimulation or out control of inflammation accelerated the proliferation of tumor cells.Tumor-associated inflammation promotes the proliferation and survival of tumor cells [44], which agrees with our experimental results that the HTBS group experienced the fastest tumor growth, and the QDBS group had the most obvious lung metastasis. In TCM, deficiency of Zhengqi refers to low immunity and is the fundamental cause of tumor metastasis [45, 46]. If Zhengqi is weak, cancer toxins are insidious and will cause gas stagnation, blood stasis, wet fusion, phlegm coagulation, poison fusion, and finally the spread of cancer to other organs [47].

## 5. Conclusions

**Figure 6:**
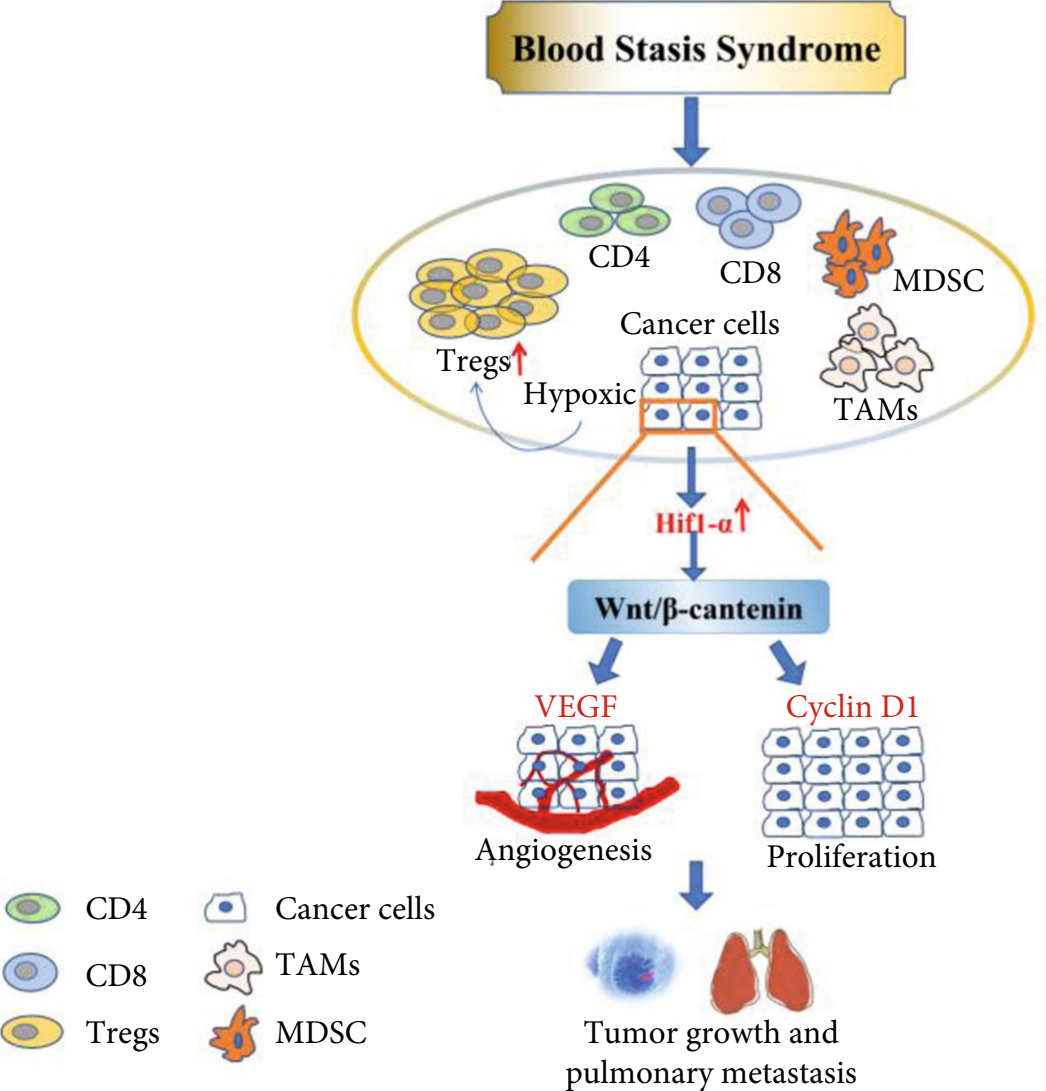
Schematic showing BBS mechanisms involved in regulating hypoxia and immunosuppressive microenvironment in breast cancer. By aggravating hypoxia tumor microenvironment, BSSs promote HIF1-*α* expression, thus upregulating VEGF and Cyclin D1 through the Wnt/*β*-catenin signaling pathway, which promoted tumor angiogenesis; additionally, HIF1-*α* promotes the immunosuppressive microenvironment formation, thereby promoting the growth and metastasis of tumor cells.

Our study demonstrated that BSSs promoted the growth and metastasis of breast cancer, and the underlying mechanism may be related to the upregulation of VEGF and Cyclin D1 expression through the HIF1-*α*/Wnt/*β*-catenin signaling pathway, which accelerated the formation of hypoxia and the immunosuppressive microenvironment, thus promoting the growth and metastasis of tumor cells (Figure 6). Our findings provide original insights into the effect and mechanism of BSSs on breast cancer, and we expect further research to be conducted to clarify the more detailed mechanisms of this phenomenon.

## Data Availability

The original contributions presented in the study are included in the article; further inquiries can be directed to the corresponding author.
